# The Depolarizing Action of GABA in Cultured Hippocampal Neurons Is Not Due to the Absence of Ketone Bodies

**DOI:** 10.1371/journal.pone.0023020

**Published:** 2011-08-19

**Authors:** Jaylyn Waddell, Jimok Kim, Bradley E. Alger, Margaret M. McCarthy

**Affiliations:** Department of Physiology, University of Maryland School of Medicine, Baltimore, Maryland, United States of America; Sackler Medical School, Tel Aviv University, Israel

## Abstract

Two recent reports propose that the depolarizing action of GABA in the immature brain is an artifact of *in vitro* preparations in which glucose is the only energy source. The authors argue that this does not mimic the physiological environment because the suckling rats use ketone bodies and pyruvate as major sources of metabolic energy. Here, we show that availability of physiologically relevant levels of ketone bodies has no impact on the excitatory action of GABA in immature cultured hippocampal neurons. Addition of β-hydroxybutyrate (BHB), the primary ketone body in the neonate rat, affected neither intracellular calcium elevation nor membrane depolarizations induced by the GABA-A receptor agonist muscimol, when assessed with calcium imaging or perforated patch-clamp recording, respectively. These results confirm that the addition of ketone bodies to the extracellular environment to mimic conditions in the neonatal brain does not reverse the chloride gradient and therefore render GABA hyperpolarizing. Our data are consistent with the existence of a genuine “developmental switch” mechanism in which GABA goes from having a predominantly excitatory role in immature cells to a predominantly inhibitory one in adults.

## Introduction

GABA is the primary inhibitory neurotransmitter in the mature brain. However, in immature cells, GABA induces cell membrane depolarization by opening of the GABA-A receptor channel, a chloride-permeable ionophore [1]. GABA is assumed to provide the primary excitatory drive for neuronal networks in early development, when GABAergic synapses outnumber glutamatergic synapses [2], [3]. In immature cells, GABA is excitatory because the cells express high levels of the NKCC1 cotransporter relative to the KCC2 cotransporter [4], [5]. NKCC1 pumps Cl^−^ into the cells and thereby maintains a concentration of Cl^−^ ([Cl^−^]) that is relatively high [4], [5]. The elevated intracellular [Cl^−^] establishes a transmembrane Cl^−^ equilibrium potential (E_Cl_) that is positive with respect to the resting potential. In this condition, opening of the GABA-A channels leads to an efflux of Cl^−^ and, consequently, membrane depolarization. As development progresses, activity of the KCC2 cotransporter increases, and as it extrudes Cl^−^, KCC2 reduces intracellular [Cl^−^] and establishes an E_Cl_ that is negative with respect to the resting membrane potential. In mature neurons, opening of GABA-A receptor channels allows Cl^−^ to enter the cell and hyperpolarize the membrane [5]. The progressive increase in KCC2 activity coupled with a decrease in activity of NKCC1 is commonly referred to as the developmental “GABAergic switch” in which GABA gradually becomes inhibitory as neurons mature [4], [5]. The changing pattern of expression of these cotransporters has been documented in several regions of the developing brain, and has been measured in a variety of experimental preparations. Extensive converging evidence has revealed the critical role of GABA-mediated excitation in initiation of calcium-sensitive signaling cascades that control DNA synthesis, proliferation, migration, neuronal differentiation and cell-to-cell communication [6], [7], [8], [9], [10], [11], [12], [13].

Nevertheless, two recent reports suggest that the depolarizing action of GABA is an artifact of insufficient metabolic substrate availability *in vitro* [14], [15]. In vitro preparations typically include only glucose as an energy source. It was proposed that this is not likely to mirror metabolic substrate availability in suckling neonatal rat pups, as breast milk is low in carbohydrate and high in fat, which leads to ketosis [e.g., 16]. Thus, in the neonatal rat, ketone bodies (especially β-hydroxybutyrate, BHB), as well as pyruvate and lactate, would comprise the main pool of available energy substrates [16], [17], [18], [19]. Deficiency of ketones reportedly leads to inadequate Cl^−^ transport and, the addition of ketone bodies to perfusates appears to stimulate pumps or cotransporters that renders GABA hyperpolarizing, even in immature neurons *in vitro* [14], [15]. The authors conclude that glucose alone cannot provide sufficient energy for mitochondrial respiration in immature neurons, and that therefore the occurrence of depolarizing GABA-A receptor responses reflects an artifact of an insufficient energy supply. Recently this hypothesis has been strongly challenged [20], [21], [22] however.

The proposal that the depolarizing action of GABA is caused by insufficient energy substrate has profound implications for the study of brain development. Our laboratory has demonstrated robust sex differences in the temporal progression of GABA action from depolarizing to hyperpolarizing in the developing brain [e.g.], [ 23,24]. Our results parallel those of other labs in showing that sex differences in GABA-mediated excitation are mediated by gonadal steroids [e.g. 7], [24], [25], [26]. If the existence of depolarizing GABA-A receptor responses is not well founded, then the mechanisms of sex-differences in brain development would have to fundamentally reevaluated.

In most of the recent studies [20], [21], [22] acute hippocampal slices from very young tissue were used. Glucose utilization rates rise in young tissue subjected to trauma such as seizures, however [27], and it seemed possible that there might be a greater reliance on glucose in cut tissue such as acute slices, which could mask a dependency on ketone bodies in young tissue. Accordingly, we have re-examined the issue of energy substrates in dissociated cultures of immature hippocampal neurons that have recovered from the isolation procedures and might therefore be more dependent on ketone bodies as their energy source. We tested the basic predictions of the inadequate energy supply hypothesis [14], i.e., whether addition of the energy substrate BHB to experimental solutions (i.e., media and perfusates) would abolish depolarizing GABA responses measured by calcium imagining or perforated patch-clamp techniques. Nevertheless, we find that the addition of BHB did not affect the depolarizing action of GABA assessed with either measure of excitation in cultured cells. Our data are therefore consistent with the recent findings against the ketone body hypothesis, and agree with the concept that depolarizing GABA-A receptor responses are physiologically relevant phenomena. Other factors, such as acidosis, may have contributed to the effects reportedly due to energy deprivation [14], [15; see 21 for discussion].

## Materials and Methods

### Cell Culture

Newborn (postnatal day 0) male and female rats (Long Evans) were obtained from breeder females at the University of Maryland, Baltimore. Animal use procedures were approved by the University of Maryland, Baltimore IACUC (Protocol Number: 0708007), and followed NIH guidelines. Hippocampi were dissected into Hank's balanced salt solution (HBSS+; Ca^2+^ and Mg^2+^-free) supplemented with 10 mM HEPES (pH 7.3) and antibiotic/antimycotic, then additional HBSS+ was added to the tube to a volume of 4.5 ml, with 0.5 ml trypsin (2.5%), and incubated for 15 min at 37°C. Supernatant was discarded and tissue was washed twice with HBSS+, dissociated by trituration, plated on 25 mm poly-l-lysine coated cover slips at a density of 300,000 cells per coverslip, and placed in 100 mm dishes containing 4 ml plating medium (MEM supplemented with 10% horse serum, 0.6% glucose and 1 mM pyruvate). Cell number and viability were determined by trypan blue exclusion and cells were allowed a minimum of 4 h to adhere to the coverslips in a 37°C, 5% CO_2_ incubator after which coverslips were removed from the plating dishes and placed into 35 mm dishes filled with Neurobasal medium supplemented with 2% B-27, antibiotic/antimycotic, 0.5 mM l-glutamine.

### Ca^2+^ imaging

Hippocampal neurons were incubated for 20 min at 37°C with one of three physiological salt solutions (PSS) containing the respective metabolic substrate (all concentrations in mM): 1) Standard PSS: NaCl 134, glucose 25, KCl 5, MgCl_2_ 1, CaCl_2_ 3; NaHCO_3_ 5; 2) Standard PSS (with glucose 25) plus BHB 4; 3) Standard PSS without glucose, with BHB 4 as the only metabolic substrate. BHB was supplied by Sigma Aldrich. The pH was maintained at 7.4 using HEPES with the exception of two conditions referred to as “Low pH.” In these two conditions, the pH of PSS containing glucose or BHB was lowered to 7.1 with HCl-. Following this incubation, each cover slip was incubated for 20 min more following application of the cell-permeant fluorescent calcium indicator Fura-2-AM (3 µM; Molecular Probes, Eugene, OR, USA) in DMSO (0.5%), rinsed with PSS and incubated for an additional 10 min. Thus, each cover slip was treated with PSS containing either glucose, BHB or both for at least 50 min prior to data collection. Cover slips were then transferred to a chamber mounted on a microscope stage, and superfused with the respective PSS at 32–34°C. The imaging system consisted of a Zeiss Axiovert 100 inverted microscope with TILL Photonics Polychrome II Monochromator (Applied Scientific Instrumentation, Eugene, OR, USA), a Hamamatsu CCD video camera and image intensifier. Image acquisition and analysis were performed with Metamorph/Metafluor Imaging System, version 5.0 (Universal Imaging Corporation, Downingtown, PA, USA). Ratiometric fluorescence imaging data (excitation at 340 and 380 nm) were collected every 10 sec at 510-nm emission wavelength to calculate an F340/F380 fluorescence ratio that is used to monitor changes in intracellular [Ca^2+^] ([Ca^2+^]_i_) Cells in the field of view were characterized morphologically using a 60× objective, enabling us to distinguish between neurons and glia. Cells characterized by at least two primary processes were selected for data collection. Cells amorphous in shape, with no distinct processes were judged to be glia, and were not selected for data collection. Individual cells chosen for analysis were traced using the Metafluor program.

### Electrophysiology

Perforated patch-clamp recordings were made at 22–24°C in a bath solution consisting of (in mM) 126 NaCl, 3 KCl, 2 CaCl_2_, 2 MgSO_4_, 26 NaHCO_3_, 1 NaH_2_PO_4_, 12 glucose, 0.0005 tetrodotoxin (TTX, voltage-gated sodium channel blocker) and 0.01 nifedipine (L-type voltage-gated Ca^2+^ channel blocker) (bubbled with 95% O_2_/5% CO_2_). d,l-BHB (4 mM) was freshly added to the bath solution within 8 hr of recording. In control experiments, BHB was replaced by 6 mM sucrose to maintain equal osmolarity (300–305 mOsm). Before testing the effect of muscimol, we incubated cells in BHB-containing bath solution for 51–109 min at 22–24°C. The bath solution was perfused at a rate of ∼1.5 ml/min. The recording pipette solution contained (in mM) 140 KCl, 5 NaCl, 10 HEPES, 0.5 CaCl_2_, 5 EGTA, 50 µg/ml gramicidin (pH 7.20 with KOH, 295 mOsm). The electrode tip was filled with gramicidin-free pipette solution to facilitate seal formation. Gramicidin stock solution (50 mg/ml in DMSO) was added to the pipette solution <1 hr before recording. Data were collected using an Axopatch 200B amplifier (Molecular Devices, Sunnyvale, CA), and were filtered at 1 kHz and digitized at 10 kHz in Clampex 9 (Molecular Devices). Series resistance reached steady state values (56±9 MΩ in 5 control cells and 61±12 MΩ in 5 BHB-treated cells; p>0.7, t-test) within 15–50 min after gigaseal formation. To ensure that inadvertent break-in to whole-cell mode did not occur after perforated patch establishment, we carefully monitored series resistance. Break-in to whole cell mode would be accompanied by a sudden drop in series resistance to ∼10 MΩ with these electrodes, and as noted, our final series resistances were considerably higher and stable during the experiment. In addition, with our total pipette [Cl^−^] of ∼146 mM, E_GABA_ would be at ∼0 mV membrane potential in whole-cell mode, however even with maximal muscimol responses, we never observed depolarizations more than to ∼−30 mV. Finally, we observed that break-in to whole-cell mode with these electrodes quickly led to drastic changes in cell morphology, which did not happen when in perforated patch mode. These checks were applied to all perforated patch recordings.

## Results

Hippocampal neurons were cultured from pups on the day of birth and Ca^2+^ imaging was conducted on days 1–3 *in vitro* (DIV) following a previously published protocol [24], [25]. A total of 23 coverslips from two independent cultures were sampled. Baseline measurement of resting [Ca^2+^]_i_ for individual cells was obtained over a 5-min period while the cells were superfused with PSS. After 5 min, a 50-sec pulse of muscimol (10 µM) was delivered, and data was acquired for an additional 5 to 7 min until baseline was re-established. BHB was added to replicate the solutions reported by Rheims et al. [14] to reverse the depolarizing action of GABA. The third perfusate excluded glucose to determine whether BHB would be sufficient to support cell metabolism. The following parameters were calculated from cells exhibiting at least a 10% increase over baseline in response to muscimol: 1) average baseline [Ca^2+^]_i_ and 2) peak [Ca^2+^]_i_ following muscimol application 3) percentage of cells responding.

**Figure 1:**
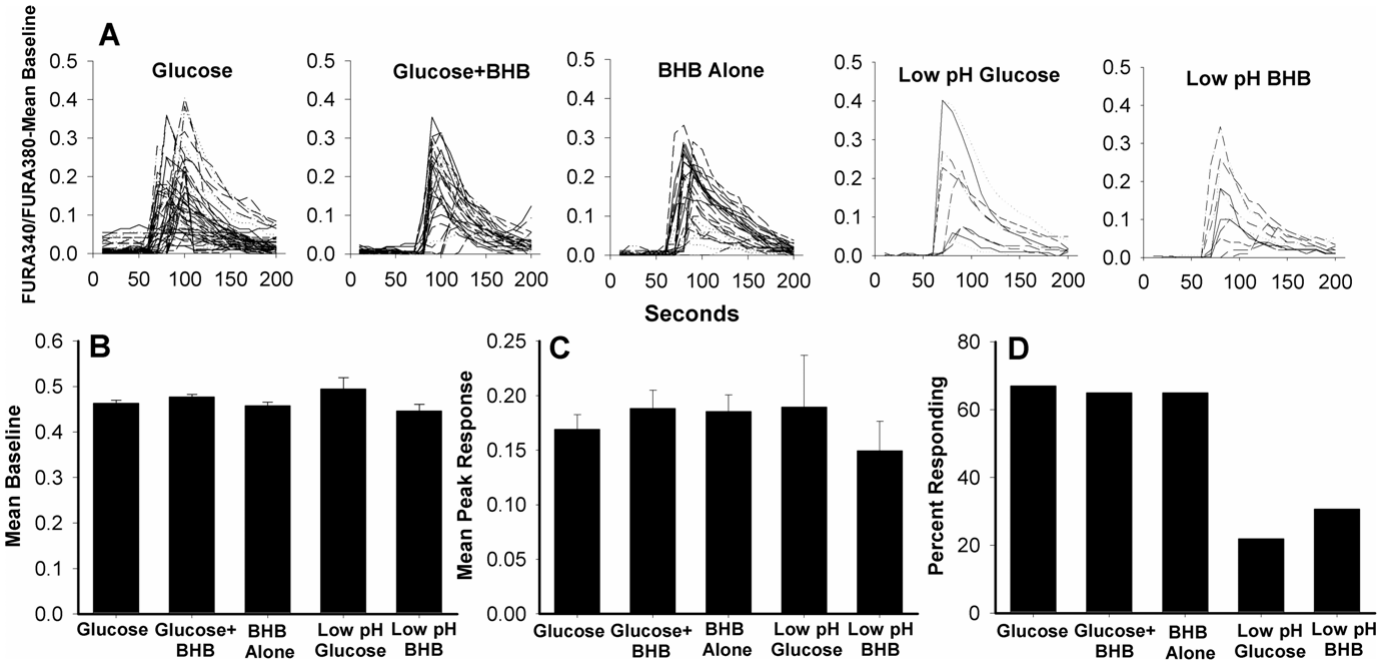
Muscimol-induced calcium influx in BHB-treated hippocampal cultured neurons. A. Traces of muscimol-induced calcium responses in immature dispersed hippocampal neurons with glucose alone, ketone bodies (BHB) with glucose, or BHB alone. The last two top panels are traces from cells tested with experimental solutions with a lower pH. Addition of BHB to perfusates did not reduce muscimol-induced calcium influx. B. Ketone bodies did not change the average baseline or C. the average peak in [Ca2+]I in response to the GABA-A agonist muscimol. Error bars represent the standard error of the mean. D. Percentage of cells responding in each condition. Lowering the pH significantly decreased the number of cells exhibiting excitatory responses to muscimol.

Addition of BHB did not impact the depolarizing action of muscimol as indexed by Ca^2+^ entry. Traces of responding cells are presented in Figure 1A for each perfusate condition. One-way ANOVA on energy substrate×peak [Ca^2+^]_i_ indicated no significant difference between groups F(4, 128) = 1.62, p = .173 (Figure 1C). Therefore, we infer that the addition of BHB to the perfusate did not attenuate the depolarizing response to the GABA-A receptor agonist muscimol. The addition of BHB also did not impact the average baseline in each condition F(4,128)<1.00, p = .5 (Figure 1B). Finally, BHB did not change the percentage of neurons responding to muscimol in the three conditions in which the pH of experimental solutions was 7.4. For each of these three conditions, the number of cells sampled and the number of cells exhibiting excitatory responses to muscimol were: Standard PSS = 67% (52 of 78 cells); PSS+Glucose+BHB = 65% (30 out of 46 cells); PSS+BHB Alone (no glucose) = 65% (30 out of 46 cells). Reducing the pH resulted in a robust drop in the number of cells responding to muscimol irrespective of metabolic substrate (Figure 1D): Standard PSS = 21.9% or 9 out of 41 cells; PSS+Glucose+BHB = 30% or 12 out of 39 cells. This reduction followed addition of HCl to the perfusate, bringing the pH to 7.1. Reducing the pH produced a significant reduction in the likelihood of excitatory responses, χ^2^ (1, N = 251) = 32.99, p = .0001. These data therefore suggest that our BHB treatment did not alter pH in our experiments.

Ca^2+^ influx initiated by depolarizing GABA-A responses largely occurs through voltage-gated Ca^2+^ channels [e.g., 26], hence our results showing that BHB did not alter Ca^2+^ influx, argue that GABA-A responses in BHB were capable of depolarizing the cells sufficiently to open voltage-gated Ca^2+^ channels. However, it was conceivable that the GABA-A depolarization might be much reduced but still capable of opening the Ca^2+^ channels, or even that the Ca^2+^ channels themselves were altered in some way that enabled them to open at hyperpolarized potentials.

**Figure 2:**
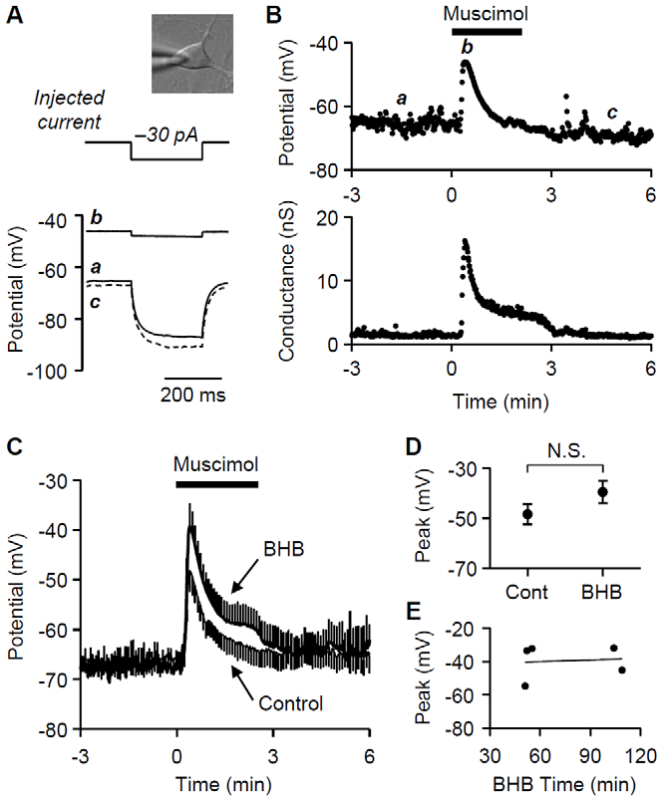
Muscimol-induced depolarization in BHB-treated hippocampal cultured neurons. **A**. In current clamp mode with gramicidin-perforated patch clamp, the membrane potential of a representative hippocampal neuron was recorded with repetitive injection of −30 pA step currents (250 ms duration) at 1 Hz. The voltage traces were sampled at the indicated time points of the graph in B. Inset, DIC image of a putative pyramidal neuron. **B**. Data from a representative control cell. The upper graph shows resting membrane potential measured at the baseline before the injection of −30 pA current. The lower graph is a plot of membrane conductance calculated from the maximum voltage deflection during the −30 pA injection. Muscimol (10 µM) was bath applied for 2 min. **C**. The averaged data show that muscimol (10 µM; 2–3 min) depolarized both BHB-treated (n = 5) and control (n = 5) cells. Error bars (S.E.M.) were plotted only every 5 s for clarity. **D**. The peak membrane potential during muscimol application was averaged over 5 s, and compared between control and BHB-treated neurons. N.S., not significant. **E**. From the BHB-treated cell, the peak membrane potential in muscimol was plotted against the duration of BHB treatment. The straight line is linear regression, *y* = 0.03*x*−42 (R^2^ = 0.008).

To test these possibilities, we recorded muscimol-induced changes in the membrane potentials of cultured hippocampal neurons at 3–11 DIV, while preserving the endogenous [Cl^−^] with a perforated patch-clamp technique. Putative pyramidal cells were selected based on somatic morphology (Figure 2A). We recorded the resting membrane potentials using a gramicidin-perforated patch clamp technique while monitoring membrane conductance with voltage responses to small step current injections (−10 to −30 pA, 250 ms) at 1 Hz (Figure 2AB). In control conditions (i.e., absence of BHB) the cells had resting potentials of −66±3 mV and input conductances of 1.06±0.22 nS. Muscimol (10 µM), which was applied to the flowing bath solution for 2–3 min, transiently depolarized the membrane potential of control cells by ∼18 mV (to −48±4 mV; n = 5) (Figure 2B), and increased the membrane conductance (to 13.6±2.1 nS) with a time course similar to that of the change in membrane potential (Figure 2B). Because TTX and nifedipine were present in the bath solution, the increases in membrane potential and conductance reflected direct effects of muscimol on membrane properties, and were not secondary effects of opening Na^+^ and Ca^2+^ channels.

We observed that, unlike the previous report [12], 10 µM muscimol also depolarized BHB-treated neurons in our conditions (Figure 2C). Cells treated with BHB for 51–109 min had resting potentials of −67±3 mV and input conductances of 1.49±0.39 nS (n = 5), i.e., indistinguishable from control values (p>0.4, t-tests). In these cells, muscimol induced a peak depolarization (averaged over 5 sec) of ∼27 mV, to −40±5 mV which was not significantly different from the depolarization of control cells (p>0.05, t-test; Figure 2CD). Similarly the muscimol-increased the input conductance (to 14.4±1.7 nS), did not differ from the control value (p>0.7, t-test). The inability of BHB to abolish the muscimol-induced depolarization, i.e., to reduce intracellular [Cl^−^] in immature neurons, implies that BHB might have failed to supply sufficient energy to neurons, according to the previous interpretation [14]. Although our BHB treatment time was comparable to what was used previously [14], it is possible that a longer BHB treatment could supply more energy to be used to reduce intracellular [Cl^−^]. In this case there should be a relationship between the duration of BHB treatment and the reduction in the muscimol response. We therefore asked if the duration of BHB treatment was correlated with the magnitude of muscimol-induced depolarization, but found no correlation between them (Figure 2E), suggesting that the duration of BHB supply in our condition was not a determining factor. Thus, we do not find evidence that the depolarizing action of GABA reflects the absence of adequate substrate for mitochondrial respiration.

## Discussion

Our data support the conclusion that depolarizing GABA-A receptor responses are genuine phenomena in immature neurons, and not artifacts of experimental conditions. The results do not support the hypothesis that the depolarizing action of GABA observed in cultured neurons is due to deprivation of adequate energy substrates. Though ketone bodies do supply a significant proportion of energy to the developing brain [16], provision of exogenous ketone bodies to immature neurons *in vitro* does not change muscimol-induced excitation as measured by Ca^2+^ influx or perforated patch-clamp electrophysiology. Our experiments on cultured cells therefore complement other recent reports demonstrating that the addition of lactate or BHB does not render GABA hyperpolarizing in slice preparations of hippocampal and neocortical cells [20], [21], [22]. Thus, glucose appears to be sufficient to support cellular energy demands across a variety of *in vitro* preparations.

We also note that, though Rheims et al. [14] and Holmgren et al. [15] highlight a potentially important shortfall of *in vitro* preparations, their proposal fails to account for three well documented phenomena concerning GABA-mediated excitation. First, analysis of the developmental time courses of expression of the cotransporters that modulate the chloride gradient, NKCC1 and KCC2, yields the same results in preparations that are not likely to suffer from energy deficiency. For instance, western blotting has confirmed a “developmental switch” in NKCC1 and KCC2 protein using tissue extracted and frozen quickly from nursing rat pups [e.g.], [ 19], [23], [25,28]. Further, premature expression of KCC2 *in vivo* reduces the length of dendrites and the number of dendritic branches, suggesting that the depolarizing action of GABA shapes synaptic connections in the developing brain [29]. Second, immature cells in the adult brain exhibit excitatory responses to GABA, a time at which glucose is the primary source of energy in the brain [16], [30], [31], [32], [33]. Third, injury can induce depolarizing responses to GABA in the adult brain, suggesting that this developmental program is reactivated [34], [35], [36] and not that the adult brain switches to a different energy source. Thus, it appears that depolarizing GABA comprises an obligatory phase of neuronal development, irrespective of the age of the animal, or the primary source of energy, which is glucose in the adult brain [16].

One proposed alternative explanation for the results of Rheims et al. [14] and Holmgren et al. [15] is that they reflect responses to acidosis caused by the addition of weak acids, such as BHB [21], [22]. Treatment with weak acids, including propionate or lactate, reduced the frequency of giant depolarizing potentials (GDPs) elicited by GABA, although the GDPs eventually recovered in the presence of propionate or lactate. Application of the poorly metabolized lactate, d-lactate, similarly suppressed GDPs [21]. We used the same concentration of ketone bodies reported by Rheims et al. [14], and failed to see even a slight attenuation of the depolarizing action of GABA, unless we directly manipulated the pH of experimental solutions. Reducing the pH of experimental solutions reduced significantly the number of cells exhibiting calcium transients in response to muscimol. Tyzio et al. [22] also reported that the commercial source of BHB determined the impact of ketone bodies on GABA-induced depolarizations. Ketone bodies supplied by Acros Organics but not Sigma Aldrich attenuated the depolarizing action of GABA; ketone bodies obtained from Acros reportedly also contained contaminants, which alone were sufficient to disrupt GABA-induced depolarizations in immature neurons [22]. The ketone bodies used here were obtained from Sigma Aldrich, and are therefore likely to be free of contaminants. Finally, contrary to the previous proposals [14], [15] it appears that deprivation of energy substrate does not render developing cells hyperexcitable. If it did, then the absence of energy substrate should support GABA-mediated depolarizations, yet this was not the case, as removal of all energy substrates, including glucose, suppresses GDPs [21]. The major implication of this work is that depolarizing GABA-A receptor responses represent a vital excitatory drive in the developing CNS and can profitably be investigated *in vitro*.
